# Hospitalization and mortality during the pandemic in chronic hemodialysis patients and the general population in Peru

**DOI:** 10.1590/2175-8239-JBN-2022-0149en

**Published:** 2023-05-15

**Authors:** Percy Herrera-Añazco, Moisés Apolaya Segura, Jessica Bravo-Zúñiga, Juan Lluncor Vásquez, Alvaro Taype-Rondán

**Affiliations:** 1 Universidad Privada del Norte, Trujillo, Peru.; 2 Red Peruana de Salud Colectiva, Lima, Peru.; 3 Universidad César Vallejo, Trujillo, Peru.; 4 Hospital Nacional Edgardo Rebagliati Martins, Departamento de Nefrología, Lima, Peru.; 5 Centro Nacional de Salud Renal, EsSalud, Lima, Peru.; 6 Universidad San Ignacio de Loyola, Unidad de Investigación Para la Generación y Síntesis de Evidencias en Salud, Lima, Peru.

**Keywords:** Dialysi, Coronavirus infection, Hospitalizatio, Mortalit, Peru, Diálise, Infecções por Coronavírus, Hospitalização, Mortalidade, Peru

## Abstract

**Background:**

Patients with chronic kidney disease have a higher risk of severe disease and mortality from COVID-19 than the general population.

**Objective:**

To compare hospitalization and mortality rates during the pandemic among chronic hemodialysis (HD) patients and the general population in Lima (Peru).

**Methods:**

This retrospective cohort included an assessment of the database of chronic HD patients of the health service providers of the social health insurance benefit networks of Lima and Callao between 2019 and 2021. Hospitalization and mortality rates were obtained for every 1,000 individuals, and variations in the percentages of COVID-19 cases and deaths were calculated. These rates were compared with those of the general population data and standardized by age and sex.

**Results:**

An average of 3,937 chronic HD patients were evaluated each month. Of these, 4.8% had COVID-19 and 64.97% were mild cases. The hospitalization rates per 1,000 patients were 19.5, 29.28, and 36.7 in 2019, 2020, and 2021, respectively. The mortality rates per 1,000 patients were 5.9, 9.74, and 11.49 in 2019, 2020, and 2021, respectively. When compared to the standardized general population, the peaks of both rates coincided with the plateaus of the waves during the pandemic. The hospitalization rate for COVID-19 was 12 times higher in HD patients than in the general population, and the mortality rate for COVID-19 was twice as high.

**Conclusion:**

HD patients had higher hospitalization and standardized mortality rates than the general population. Peaks in hospitalizations and mortality coincided with the plateaus of the first and second waves of the pandemic.

## Resumo

**Histórico:**

Pacientes com DRC apresentam maior risco de doença grave e mortalidade por COVID-19 do que a população geral.

**Objetivo:**

Comparar taxas de hospitalização e mortalidade durante a pandemia entre pacientes em hemodiálise crônica (HD) e a população geral em Lima (Peru).

**Métodos:**

Esta coorte retrospectiva incluiu avaliação do banco de dados de pacientes em HD crônica dos prestadores de serviços de saúde das redes de benefícios do seguro social de saúde de Lima e Callao, entre 2019–2021. Obteve-se taxas de hospitalização e mortalidade para cada 1.000 indivíduos, e foram calculadas variações nas porcentagens de casos de COVID-19 e óbitos. Estas taxas foram comparadas com os dados da população geral e padronizadas por idade e sexo.

**Resultados:**

Uma média de 3.937 pacientes em HD crônica foram avaliados mensalmente. Destes, 4,8% tinham COVID-19, 64,97% eram casos leves. As taxas de hospitalização por 1.000 pacientes foram 19,5; 29,28; e 36,7 em 2019, 2020, e 2021, respectivamente. As taxas de mortalidade por 1.000 pacientes foram 5,9; 9,74 e 11,49 em 2019, 2020, e 2021, respectivamente. Quando comparados à população geral padronizada, os picos das taxas coincidiram com os platôs das ondas da pandemia. A taxa de hospitalização para COVID-19 foi 12 vezes maior em pacientes em HD do que na população geral e a taxa de mortalidade por COVID-19 foi duas vezes maior.

**Conclusão:**

Pacientes em HD apresentaram taxas de hospitalização e mortalidade padronizada mais elevadas do que a população geral. Os picos das hospitalizações e mortalidade coincidiram com os platôs da primeira e segunda ondas da pandemia.

## Introduction

Although the distribution of the COVID-19 vaccine reduced severe cases and mortality from the disease, the pandemic remains a public health concern because of new variants of the virus and gaps in vaccine coverage^1,2,3,4^. According to the World Health Organization, there were 555 million confirmed cases and more than six million deaths worldwide at the beginning of July 2022^5^.

Patients with chronic kidney disease (CKD) have a higher risk of severe disease and mortality from COVID-19 than the general population^6^. Some studies suggest that the incidence and mortality associated with COVID-19 are higher in hemodialysis (HD) patients than in patients with CKD who do not require HD^6,7^.

The effect of the disease on dialysis centers worldwide varied depending on the country of study^8^. Therefore, the number of patients who did not receive HD during the pandemic was higher in low- and middle-income countries than in high-income countries^9^. Similarly, HD patients in low- and middle-income countries had lower access to intensive care units and mechanical ventilation than patients in high-income countries^9^.

COVID-19 incidence and mortality in HD patients varied as the pandemic progressed. A study conducted in Poland from the beginning of the pandemic to January 2021 found that the increase and decrease in the number of new cases occurred first in patients undergoing HD, although the epidemic trajectory was parallel in HD patients and the general population^10^. Likewise, there is a discrepancy in patient prognosis based on the period evaluated, with some studies indicating that mortality was higher in the second wave than in the first^11^, while others found no differences^12^.

According to some studies, Peru is a middle-income country with one of the highest COVID-19 mortality rates^13^. This was due to factors such as a fragmented health system and a lack of coordination among the different levels in charge of managing the pandemic^14^. Although some reports showed the effect of the pandemic in chronic HD centers, these were single-center reports and not representative of the national picture^15^. Similarly, although these patients have a worse prognosis compared to the general population, no standardized comparisons have been made in Peru in this regard^6,7^.

Our study aimed to compare COVID-19 hospitalization and mortality rates among chronic HD patients with social health insurance (EsSalud) and the general population in Lima (Perú), considering that the impact of the pandemic in HD patients varies according to the country of origin^8,9^.

## Material and Methods

### Design and Population

A retrospective cohort study was conducted comparing chronic HD patients treated at EsSalud with the general population of Lima (Peru).

EsSalud is an institution that cares for approximately 30% of the population at the national level, with service networks that have health service providers (IPRESS) that provide HD to patients with CKD. Lima and Callao have three service networks: Rebagliati, Almenara, and Sabogal.

This study included chronic patients receiving HD who were treated at the IPRESS of the healthcare networks of the Hospital Nacional Edgardo Rebagliati Martins, Hospital Nacional Guillermo Almenara Irigoyen, and Hospital Nacional Alberto Sabogal Sologuren between January 2019 and December 2021. Patients referred to another IPRESS with an unknown final destination were excluded. The demographic and clinical characteristics of the 3,677 HD patients treated in July 2021 were described.

### Variables

The main individual variables studied were age, sex, the benefit network to which the patients belonged (Rebagliati, Almenara, or Sabogal), and the cause of CKD (diabetes mellitus, glomerulonephritis, arterial hypertension, systemic lupus erythematosus, obstructive uropathy, or non-affiliated, as established in the medical record).

Furthermore, data from the clinical history were considered, such as hepatitis infection (defined as a previous serological diagnosis of hepatitis B or C) and COVID-19 infection prior to July 2021 (confirmed either by serological or molecular test or by epidemiological criteria). Likewise, COVID-19 severity (mild, moderate, or severe) was also considered, with mild indicating that the patient did not require hospitalization, moderate indicating that the patient required hospitalization but no mechanical ventilation, and severe indicating that the patient required mechanical ventilation.

Consolidated variables per month, such as number of patients treated with HD, hospitalized for all causes, and who died from all causes, were considered. The number of patients hospitalized for COVID-19 and who died from COVID-19 was also included.

### Data Collection Procedure

After the protocol was approved by the National Center for Kidney Health, the HD patients’ demographic and clinical data were obtained from the Comprehensive System of Contracted Services platform, version 3.2, and from the vaccination registry of each IPRESS consolidated by the Office of Contracted Services. The information on hospitalization and death was obtained from the EsSalud Intelligent Health Services computer system.

For the general population, data on COVID-19 deaths were obtained from the MINSA open data website^16^. Data on deaths from all causes were obtained from the open data website of the National Death Information System^17^. Lima population data were obtained from the 2018–2020 Report on Population Estimates and Projections based on department, province, and district^18^.

After coding, all data were anonymously stored in a Microsoft Excel program sheet.

### Data Analysis

Absolute and relative frequencies for categorical variables and means and standard deviations for numerical variables were calculated.

The hospitalization and mortality rates of the general population were standardized relative to HD patients treated in July 2021. Monthly mortality rates for each sex and age group were used (every 5 years)^19^.

Hospitalization and mortality rates were obtained for every 1,000 HD patients to standardize morbidity and mortality over time. Moreover, the monthly percentage variations attributed to COVID-19 cases and deaths as well as the percentage increases over the 2019 average were calculated. Finally, line graphs were constructed to predict the behavior of morbidity and mortality rates over time.

### Ethical Aspects

The study protocol was approved by the Hospital Nacional Alberto Sabogal Sologuren Ethics Committee according to Memorandum No. 035-CIEI-OFIyD-GRPS-ESSALUD-2022.

## Results

An average of 3,937 ± 167.4 HD patients were treated every month at the EsSalud IPRESS from January 2019 to December 2021. Among HD patients in July 2021, male sex predominated (n = 1,726, 54.74%), the mean age was 59.65 ± 14.86 years, most were from the Rebagliati Network (n = 1,325, 42.05%), and the main cause of CKD was diabetes mellitus (n = 1,294, 41.04%). COVID-19 was diagnosed in 4.8% of patients, with mild cases predominating (64.97%) (Table 1). In July 2021, 3,677 HD patients were treated, of whom 327 received no dose of the COVID-19 vaccine, 197 received 1 dose, and 3,153 receiving both doses.

**Table 1.** Characteristics of patients treated in july 2021 in hemodialysis centers of lima in ipress of essalud

Variable		N	%
Sex	Female	1427	45.26%
	Male	1726	54.74%
Age (years)	Mean, SD	59.65	14.86
Healthcare network	Rebagliati	1325	42.05%
	Beacon	1178	37.38%
	Sabogal	648	20.56%
Cause of CKD	Mellitus diabetes	1294	41.04%
	Unknown	834	26.45%
	Arterial hypertension	572	18.14%
	Obstructive uropathy	210	6.66%
	Glomerulonephritis	188	5.96%
	Lupus erythematosus	55	1.74%
Hepatitis C infection	Yes	54	1.71%
	No	3099	98.29%
Prior COVID-19 infection	No	2996	95.02%
	Yes	157	4.98%
Type of COVID-19 infection	Mild	102	64.97%
	Moderate	54	34.39%
	Severe	1	0.64%

In 2019, the average hospitalization rate for HD patients was 19.5 per 1,000 patients, which increased to 29.28 per 1,000 patients in 2020 and 36.74 per 1,000 patients in 2021. COVID-19 hospitalizations accounted for up to 31.25% and 37.86% of total hospitalizations during 2020 and 2021, respectively. When the average hospitalizations from 2020 to 2021 were compared with those of 2019, hospitalizations from any cause increased by 100% in the third quarter of 2020 and by 122.27%% in the last quarter of 2021 (Table 2).

**Table 2.** Evaluation of the hospitalization and mortality rates per quarter among patients receiving hemodialysis at the ipress of essalud during 2019–2021

Year	Quarter		Hospitalization				Mortality			
		No. of patients with HD	Hospitalization rate due to any cause x 1000	Hospitalization rate due to COVID x 1000	% Contribution of COVID to hospitalization	% Increase in hospitalization compared to 2019	General mortality rate x 1000	Mortality rate due to COVID x 1000	% Contribution of COVID to mortality	% Increase in mortality compared to 2019
**2019**	I	4025	18.22	0.00	0.00%	0.00%	4.80	0.00	0.00%	0.00
	II	4113	21.23	0.00	0.00%	0.00%	6.40	0.00	0.00%	0.00
	III	4159	20.44	0.00	0.00%	0.00%	7.05	0.00	0.00%	0.00
	IV	4163	18.09	0.00	0.00%	0.00%	5.28	0.00	0.00%	0.00
	Average	4115	19.50	0.00	0.00%	0.00%	5.89	0.00	0.00%	0
**2020**	I	3994	17.94	0.17	0.93%	–7.99%	4.42	0.00	0.00%	–36.51%
	II	4012	32.82	9.31	28.35%	68.29%	14.87	7.06	47.49%	113.49%
	III	3811	39.18	12.24	31.25%	100.90%	10.41	9.88	94.96%	49.39%
	IV	3849	27.19	4.42	16.24%	39.43%	9.27	1.04	11.21%	33.01%
	Average	3916	29.28	6.53	19.19%	50.16%	9.74	4.50	38.41%	39.85%
**2021**	I	3914	23.85	9.03	37.86%	22.28%	13.63	10.30	75.63%	95.61%
	II	3731	36.99	6.70	18.12%	89.67%	13.58	7.59	55.92%	94.94%
	III	3694	42.77	1.08	2.53%	119.33%	10.47	0.18	1.72%	50.26%
	IV	3783	43.34	1.41	3.25%	122.27%	8.28	0.53	6.38%	18.88%
	Average	3780	36.74	4.56	15.44%	88.39%	11.49	4.65	34.91%	64.92%

In 2019, the average overall mortality rate in HD patients was 5.9 per 1,000 patients, which increased to 9.74 per 1,000 in 2020 and 11.49 per 1,000 patients in 2021. During those years, the maximum COVID-19 mortality rate was 4.5 per 1,000 patients and 4.65 per 1,000 patients, respectively (Table 2). COVID-19 deaths increased the mortality rate of HD patients by 94.96% and 75.63% during the third quarter of 2020 and the first quarter of 2021, respectively (Table 2). When comparing it to the 2019 average, COVID-19 mortality increased by 113.49% during the second quarter of 2020, 95.61% in the first quarter of 2021, and 18% in the last quarter of 2021 (Table 2).

**Figure 1.**
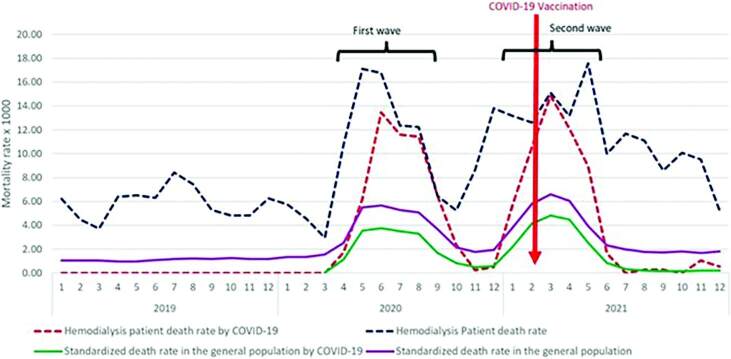
Progression of the mortality rate among hemodialysis patients at the IPRESS of EsSalud and among Lima’s standardized general population from 2019 to 2021.

The peaks of the standardized COVID-19 mortality rate in HD patients coincide with the plateaus of the first and second waves, with the second wave being higher. However, it decreased steadily after April 2021 until the end of 2021. The standardized COVID-19 mortality rate in the general population was one-third of the mortality rate in HD patients during the peaks of the first and second waves. However, both mortality rates became equal in July 2021, coinciding with the start of the COVID-19 vaccination campaign in February 2021 (Figure 1).

**Figure 2.**
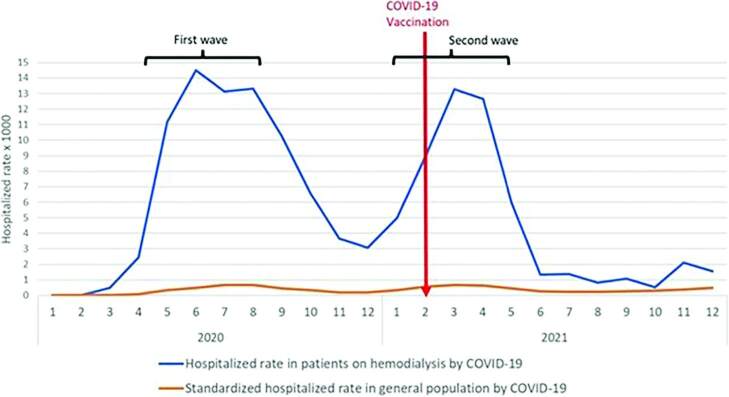
Progression of the COVID-19 hospitalization rate among hemodialysis patients in the IPRESS of EsSalud and among the standardized general population of Lima from 2020 to 2021.

The standardized hospitalization rate of HD patients increased after the pandemic began, and its peaks coincide with the plateaus of the first and second waves of the pandemic, with the second wave being lower. The standardized COVID-19 hospitalization rate in HD patients was proportionally 12 times higher than in the general population at the peaks of both waves. However, the hospitalization rate in both groups was reduced from June 2021 (Figure 2).

## Discussion

The main results of our study show that the hospitalization rate was proportionally 12 times higher in HD patients than in the general population and that the mortality rate in the general population was one-third of that in HD patients. Peaks in hospitalizations and mortality coincided with the plateaus of the first and second waves of the pandemic, with the second wave having a higher mortality.

Despite evidence that COVID-19 incidence and mortality in patients with CKD are high, few studies have compared the impact of the pandemic on these patients with the general population^6,7^. A study in Poland showed that the epidemic trajectory was similar in patients in the general population and HD patients, but the increase and decrease in the number of new cases occurred first in HD patients^10^. Although our study did not evaluate the incidence of cases, we determined that hospitalizations and mortality from COVID-19 were higher in HD patients than in the general population. These results are not surprising because CKD is a risk factor for mortality and infection severity, with patients with severe COVID-19 necessitating hospitalization. A systematic review of 20 cohort studies showed that patients with CKD had a significantly higher risk of severe disease than patients without CKD, with a pooled odds ratio (OR) of 2.15 (95% confidence interval [CI], 1.16–4.01) (I_2_= 41; p = 0.02)^20^. Another recent systematic review of 29 studies determined that patients with severe COVID-19 were more likely to have CKD as a comorbidity (pooled OR, 1.70; 95% CI, 1.21–2.40; p = 0.002)^21^. Another systematic review showed that patients with CKD had a higher risk of death than patients without CKD, with a combined OR of 5.58 (95% CI, 3.27–9.54) (I_2_= 0; p < 0.00001)^20^.

Both standardized hospitalization and death rates for COVID-19 in HD patients coincided with the first waves of the pandemic, in which a significant proportion of hospitalizations were caused by the infection, and with stages of the pandemic in which almost all deaths were attributable to COVID-19. Although hospitalization decreased in the second wave, we observed that mortality was higher (Figures 1 and 2). Other studies assessed the progression of HD patients’ prognosis throughout the pandemic. A study in England, Wales, and Northern Ireland showed that the unadjusted survival at 28 days was similar in the first and second waves, but death occurred more quickly after infection was detected in the first wave^12^. A Swiss study and another study that used a large database of European renal replacement therapy patients showed that mortality was lower in the second wave^22,23^. Conversely, a study in Pakistan reported that more HD patients in the second wave required hospitalization (32.6% vs. 22%) or mechanical ventilation (17.4% vs. 10%) and died (28.3% vs. 20%)^11^. When comparing the mortality rate during the pandemic with the previous year, an increase of more than 100% was observed. This is similar to what was observed in the United States, where deaths among HD patients during the initial phase of the pandemic exceeded the rate predicted based on previous years’ data^24^.

The reasons why we observed lower hospitalization rates and higher mortality during the second wave compared to other studies^12,22,23^ are unclear; however, we can propose some hypotheses. Because diagnostic tests were more readily available during the second wave, cases may have been diagnosed earlier, allowing for timely management and avoiding complications that required hospitalization. Furthermore, improving case registration made it easier to identify COVID-19 deaths. Similarly, it is likely that the experience gained during the first wave optimized the medical conduct of an infected patient, thus improving epidemiological surveillance at the HD center. In contrast, an increase in self-medication as the pandemic progressed could have contributed to higher mortality, especially with the use of steroids^11^.

The higher proportion of hospitalized and deceased HD patients compared to the general population was striking, and it may be related not only to the higher risk of complications in this population but also to structural issues in the Peruvian health system during the pandemic^14^. Although specific recommendations were issued in Peru to prevent the spread of infection among HD patients^25^, they may not have been followed, as occurred with the recommendations in some Latin American countries^26^. This presumably caused an increase in cases of greater severity and subsequent mortality.

In Peru, the pandemic exceeded the capacity of health facilities at the first and second levels of care to manage mild cases^14^. Without proper management, some cases may have progressed to the point where they required hospitalization, although there is no information on this topic in Peru. A similar situation may have occurred in the treatment of complications in patients with CKD, which could explain why the general mortality of HD patients was high during the first two waves of the pandemic, even higher than deaths from COVID-19 (Figure 1).

The social immobilization measures, suspension of outpatient consultations, and prioritization of the management of infected patients that occurred in this country delayed care for other pathologies, increasing their complications^27,28^. In this sense, hospitalizations and possibly mortality from these neglected pathologies increased during the first stage of the pandemic, and HD patients were no exception. An investigation into the impact of the pandemic on patient hospitalization at the Hospital Cayetano Heredia in Lima found that out of eight patients with pathologies whose care was postponed due to the pandemic, four were affected by changes in the dialysis program, the closure of a center, a change in the scheduled shift, or transportation difficulties^27^.

Other patients experienced complications as a result of a lack of diabetes medication or a delay in cancer treatment^27^. Nationwide, primary care centers were unprepared to treat other pathologies^28^, and there was an alarming shortage of medicines^29^. Furthermore, as observed in other middle-income countries, poor adherence to the dialysis regimen because of social immobility may have influenced deaths due to complications associated with the lack of dialysis^8,9^. These structural problems, which existed before the pandemic, may explain our findings and reveal the need for profound changes in the Peruvian health system. This would not only help in dealing with cases of COVID-19 that may emerge in new waves, but also cases of other neglected pathologies.

Despite having one of the fastest growing economies in Latin America, Peru has one of the lowest rates of health investment (5.5% of the GDP in 2017)^30^. The number of beds in intensive care units was among the lowest in Latin America, implying that bed shortages were a national issue at the peak of the pandemic^31,32^. There was no profound reform in the Peruvian health system. The impact of the pandemic was devastating, and although Peru was one of the first countries to implement strict social confinement as well as safety measures and community mitigation^14,32^, large areas had only limited adherence^33^.

Vaccination against COVID-19 began in early May 2021 in EsSalud dialysis patients^34^. According to a systematic review of HD patients, 2 doses of the vaccine are effective, with a humoral response seroconversion rate ranging from 81% to 97% and no notable adverse events^35^. Even after being diagnosed with COVID-19, HD patients in India who received only 1 dose of the vaccine had a 33% lower risk of infection and a 46% lower risk of mortality in adjusted models^36^. Vaccinated HD patients in Japan had lower mortality rates and oxygen requirements than unvaccinated patients^37^. Despite vaccination, mortality among HD patients in Portugal remained higher than that in the general population^38^. We could not evaluate the effect of vaccination in HD patients in our investigation because of the short follow-up time after vaccination, but mainly because it coincided with a decrease in cases in Peru, for which an additional study is necessary to evaluate this effectiveness.

Our study has some limitations. First, it was an ecological study, thus we could not assess the phenomena studied at the individual level or control for potential confounding variables that were not listed in the clinical history. Second, our results may not be applicable to institutions other than EsSalud. Third, our results are limited to the indicated study period; new waves, new variants, and the effect of vaccination may alter the trends shown. Fourth, because of the scarcity of diagnostic tests during the first stage of the pandemic, there were cases and deaths that were not officially recorded, so some of our results may have been underestimated. Fifth, while the third dose campaign began in October 2021, the study period was unlikely to be long enough to assess its effect. However, this was a study with a sample representative of the HD population in EsSalud and the results suggests that there are structural problems in the Peruvian health system.

In conclusion, during the study period, both hospitalization and standardized mortality rates in HD patients were higher than in the general population. Peaks in hospitalizations and mortality coincided with the plateaus of the first and second waves of the pandemic.

## References

[1] Liu Q, Qin C, Liu M, Liu J (2021). Effectiveness and safety of SARS-CoV-2 vaccine in real-world studies: a systematic review and meta-analysis. Infect Dis Poverty..

[2] Sharif N, Alzahrani KJ, Ahmed SN, Dey SK (2021). Efficacy, immunogenicity and safety of COVID-19 vaccines: a systematic review and meta-analysis. Front Immunol..

[3] Vitiello A, Ferrara F, Troiano V, La Porta R (2021). COVID-19 vaccines and decreased transmission of SARS-CoV-2. Inflammopharmacology..

[4] Ciotti M, Ciccozzi M, Pieri M, Bernardini S (2022). The COVID-19 pandemic: viral variants and vaccine efficacy.. Crit Rev Clin Lab Sci..

[5] World Health Organization (2022). WHO Coronavirus (COVID-19) Dashboard [Internet].

[6] Chung EYM, Palmer SC, Natale P, Krishnan A, Cooper TE, Saglimbene VM (2021). Incidence and outcomes of COVID-19 in people with CKD: a systematic review and meta-analysis. Am J Kidney Dis..

[7] Cai R, Zhang J, Zhu Y, Liu L, Liu Y, He Q (2021). Mortality in chronic kidney disease patients with COVID-19: a systematic review and meta-analysis. Int Urol Nephrol..

[8] Aylward R, Bieber B, Guedes M, Pisoni R, Tannor EK, Dreyer G (2022). The global impact of the COVID-19 pandemic on in-center hemodialysis services: an ISN-dialysis outcomes practice patterns study survey.. Kidney Int Rep..

[9] Tannor EK, Bieber B, Aylward R, Luyckx V, Shah DS, Liew A (2022). The COVID-19 pandemic identifies significant global inequities in hemodialysis care in low and lower-middle income countries-an ISN/DOPPS survey.. Kidney Int Rep..

[10] Puchalska-Reglin´ska E, De¸bska-S´lizien´ A, Biedunkiewicz B, Tylicki P, Polewska K, Jagodzin´ski P (2021). Extremely high mortality rates among hemodialysis patients with COVID-19 before the era of SARS-CoV-2 vaccination: results from a large database from the North of Poland.. Pol Arch Intern Med..

[11] Rahim S, Dhrolia M, Qureshi R, Nasir K, Ahmad A (2022). A comparative study of the first and second waves of COVID-19 in hemodialysis patients from Pakistan. Cureus..

[12] Savino M, Santhakumaran S, Currie CSM, Onggo BSS, Evans KM, Medcalf JF (2022). Comparison of outcomes of in-centre haemodialysis patients between the 1st and 2nd COVID-19 outbreak in England, Wales, and Northern Ireland: a UK renal registry analysis. Nephron..

[13] Statista (2022). Coronavirus (COVID-19) deaths worldwide per one million population as of July 13, 2022, by country [Internet].

[14] Herrera-Añazco P, Uyen-Cateriano A, Mezones-Holguin E, Taype-Rondan A, Mayta-Tristan P, Malaga G (2021). Some lessons that Peru did not learn before the second wave of COVID-19. Int J Health Plann Manage..

[15] Herrera-Anazco P, Sanchez-Perez L, Cordova-Cueva L (2021). Prevalence, clinical characteristics, and evolution of Covid-19 infection among patients and healthcare staff of a national reference hemodialysis center in Peru. Rev Nefrol Dial. Transpl..

[16] Plataforma Nacional de Datos Abiertos (2022). Datos abiertos de COVID-19 [Internet].

[17] Plataforma Nacional de Datos Abiertos (2022). 2395 distribución de datos [Internet].

[18] Instituto Nacional de Estadística e Informática (2020). Perú: proyecciones de población, según departamento, provincia y distrito, 2018-2020 [Internet].

[19] Inskip H, Armitage P, Colton T (2005). Encyclopedia of biostatistics.

[20] Menon T, Gandhi SAQ, Tariq W, Sharma R, Sardar S, Arshad AM (2021). Impact of chronic kidney disease on severity and mortality in COVID-19 patients: a systematic review and meta-analysis. Cureus..

[21] Singh J, Malik P, Patel N, Pothuru S, Israni A, Chakinala RC (2022). Kidney disease and COVID-19 disease severity-systematic review and meta-analysis. Clin Exp Med..

[22] Guidotti R, Pruijm M, Ambühl PM (2022). COVID-19 pandemic in dialysis patients: the swiss experience. Front Public Health..

[23] Vart P, Jager KJ, Arnol M, Duivenvoorden R, Franssen CFM, Groeneveld M (2022). COVID-19 pandemic waves and mortality among patients on kidney replacement therapy.. Kidney Int Rep..

[24] Ziemba R, Campbell KN, Yang TH, Schaeffer SE, Mayo KM, McGann P (2021). Excess death estimates in patients with end-stage renal disease - United States, February-August 2020. MMWR Morb Mortal Wkly Rep..

[25] Seguro Social de Salud (2020). Clinical recommendations for the management of hemodialysis patients in the context of the Covid-19 pandemic [Internet].

[26] Herrera-Añazco P, Rabanal CL, Benites-Zapata VA (2020). Are the Latin American recommendations for the management of patients infected with COVID-19 on hemodialysis realistic in health systems with limited resources?. J Bras Nefrol..

[27] Málaga G (2020). Causas de admisión en el Hospital Cayetano Heredia durante la pandemia de COVID-19. Rev Peru Med Exp Salud Publica..

[28] Pesantes MA, Lazo-Porras M, Cárdenas MK, Diez-Canseco F, Tanaka-Zafra JH, Carrillo-Larco RM (2020). Healthcare challenges for people with diabetes during the national state of emergency due to COVID-19 in Lima, Peru: primary healthcare recommendations. Rev Peru Med Exp Salud Publica..

[29] Herrera-Añazco P, Valenzuela-Rodríguez G, Torres-Pesantes L, Toro-Huamanchumo CJ (2021). Desabastecimiento de antidiabéticos y antihipertensivos en el contexto de la etapa inicial de la pandemia por la COVID-19 en Perú. Rev Cuerpo Med HNAAA..

[30] Schwalb A, Seas C (2021). The COVID-19 pandemic in Peru: what went wrong?. Am J Trop Med Hyg..

[31] Neelsen S, O’Donnell O (2017). Progressive universalism? The impact of targeted coverage on health care access and expenditures in Peru. Health Econ..

[32] Wilder-Smith A, Freedman DO (2020). Isolation, quarantine, social distancing and community containment: pivotal role for old-style public health measures in the novel coronavirus (2019-nCoV) outbreak. J Travel Med..

[33] Fernandez-Guzman D, Soriano-Moreno DR, Ccami-Bernal F, Velasquez-Fernandez R, Morocho-Alburqueque N, De-Los-Rios-Pinto A (2022). Factors associated with prevention practices against COVID-19 in the Peruvian population: disparities between rural and urban areas. PLoS One..

[34] Seguro Social de Salud (2021). Presidenta del Seguro Social informó sobre protocolo de vacunación para pacientes renales.

[35] Mehta N, Shah S, Paudel K, Chamlagain R, Chhetri S (2022). Safety and efficacy of coronavirus disease-19 vaccines in chronic kidney disease patients under maintenance hemodialysis: a systematic review.. Health Sci Rep..

[36] Yadav AK, Sankarasubbaiyan S, Gowda Bg M, Shah K, Jha V (2021). The high mortality and impact of vaccination on COVID-19 in hemodialysis population in India during the second wave.. Kidney Int Rep..

[37] Kikuchi K, Nangaku M, Ryuzaki M, Yamakawa T, Yoshihiro O (2022). Effectiveness of SARS-CoV-2 vaccines on hemodialysis patients in Japan: a nationwide cohort study. Ther Apher Dial..

[38] Fazendeiro Matos J, Peralta R, Felix C, Pinto B, Ponce P (2022). Vaccination against COVID-19 in a network of hemodialysis units in Portugal: a promising experience. Acta Med Port..

